# Delinquentes Verhalten im Rahmen frontotemporaler Demenzen und der Alzheimer-Erkrankung

**DOI:** 10.1007/s00115-021-01070-8

**Published:** 2021-02-11

**Authors:** R. Haußmann, C. Krug, F. Noppes, M. Brandt, J. Lange, M. Donix

**Affiliations:** 1 Universitäts Demenz Centrum (UDC), Klinik und Poliklinik für Psychiatrie und Psychotherapie, Universitätsklinikum Carl Gustav Carus an der Technischen Universität Dresden, Fetscherstr. 74, 01307 Dresden, Deutschland; 2 Klinik und Poliklinik für Neurologie, Universitätsklinikum Carl Gustav Carus an der Technischen Universität Dresden, Fetscherstr. 74, 01307 Dresden, Deutschland

**Keywords:** Alterskriminalität, Delinquenz, Demenz, Schuldfähigkeitsbegutachtung, Frontotemporale Demenz, Criminal behavior of the elderly, Delinquency, Dementia, Assessment of criminal responsibility, Frontotemporal dementia

## Abstract

Seniorenkriminalität ist insgesamt ein seltenes Phänomen. Bei älteren Straftätern hat es die Justiz in hohem Maß mit Ersttätern zu tun, die mehrheitlich männlichen Geschlechts sind. Eine mögliche Ursache von Erstkriminalität im höheren Lebensalter stellen Demenzerkrankungen dar. Es ist jedoch wenig dazu bekannt, wie häufig Demenzerkrankungen tatsächlich Ursache delinquenten Verhaltens im höheren Lebensalter sind. Die Demenzprävalenz in Studien mit forensischen Kohorten älterer Straftäter ist sehr heterogen, was vor allem studienmethodisch begründet ist. Längsschnittlich begehen etwa 50 % aller Patienten mit frontotemporaler Demenz und etwa 10 % aller Patienten mit Alzheimer-Demenz Delikte im Erkrankungsverlauf. Die neurobiologischen Entstehungsmechanismen von Delinquenzverhalten im Rahmen von Demenzen sind unvollständig verstanden. Nach aktuellen Erkenntnissen wird davon ausgegangen, dass Erstdelinquenz im Rahmen von Demenzerkrankungen als Folge von Beeinträchtigungen der sozialen Kognition, Empathiefähigkeit und der Verhaltenskontrolle zu verstehen ist. Bedeutsam sind insbesondere frontale und anteriore temporale Hirnstrukturen. Demenzerkrankungen können zu Beeinträchtigungen der Schuldfähigkeit führen, weshalb forensisch-psychiatrische Sachverständige auch mit Demenzerkranken konfrontiert sind. Hierbei müssen ätiologiespezifische Besonderheiten berücksichtigt werden. Insbesondere Erstdelikte im Rahmen wesensuntypischer Persönlichkeitsänderungen nach dem 50. Lebensjahr sollten an eine neurodegenerative Ätiologie denken lassen. Insbesondere frontotemporale Demenzerkrankungen, wie die behaviorale Variante einer frontotemporalen Demenz (bvFTD), aber auch die semantische Demenz (svPPA), prädisponieren zu delinquentem Verhalten.Diese Arbeit fasst aktuelle Erkenntnisse zu dieser forensisch-psychiatrisch, aber auch klinisch relevanten Thematik zusammen.

## Abstract (en)

Criminal behavior in older people represents a rare phenomenon. Among older criminals there are many first offenders and 75% are men. Dementia poses one possible origin of delinquency in advanced age. It is unclear how often dementia is the actual cause of delinquency in older age. In studies with older criminals the prevalence of dementia was heterogeneous due to methodological study issues. In the course of the disease 50% of patients with frontotemporal dementia and 10% of patients with Alzheimer’s disease commit crimes. The neurobiological origin of delinquency in dementia is poorly understood. On the basis of current study results first delinquency in older age can be explained by impairment of social cognition, difficulties in making appropriate emotional contributions and disturbed control of behavior. Affection of frontal and anterior temporal brain structures seem to be of high relevance. As dementia impairs criminal responsibility psychiatrists are confronted with a forensic evaluation of legal culpability of older criminals. Regarding different etiologies of dementia, specific peculiarities need to be considered in a forensic psychiatric assessment. Especially frontotemporal dementia predisposes towards a wide spectrum of criminal behavior whereas patients with Alzheimer’s disease predominantly commit crimes due to cognitive impairment. The review summarizes the present knowledge about criminal behavior in the context of dementia.

## Hintergrund

Alterskriminalität wird in der vorhandenen Literatur unterschiedlich definiert, mehrheitlich aber als Kriminalität von Menschen verstanden, die 60 Jahre oder älter sind [1]. Da aber charakteristische biologische, psychologische und soziale Merkmale Älterer nicht starr von bestimmten Altersgrenzen abhängen, sind derartige Definitionen nicht unproblematisch und der Untersuchung wissenschaftlicher Fragestellungen zur Alterskriminalität nicht immer zuträglich [1]. Alterskriminalität ist ein aus gerontopsychiatrischer Sicht spannendes und beachtetes, wenngleich epidemiologisch eher randständiges Phänomen [1]. Auch wenn Psychiater qua Ausbildung wenig mit Strafrecht zu tun haben, ist die sachverständige Beurteilung der Schuldfähigkeit im Strafverfahren ein möglicher Berührungspunkt [2]. Dabei stellen Demenzerkrankungen eine mögliche Ursache krankheitsbedingter Erstkriminalität im höheren Lebensalter dar [3], weshalb sich diese Arbeit delinquentem Verhalten im Rahmen von Demenzerkrankungen widmet. Hier müssen relevante Besonderheiten Berücksichtigung finden: 1. Der Übergang von bloßem kognitivem Altern zu entsprechenden Leistungseinbußen bei Demenzerkrankungen ist fließend. Während der strafmildernde Einfluss bloßen Alterns sehr begrenzt ist, können bereits beginnende krankhafte Abbauprozesse relevante Einschränkungen der Schuldfähigkeit bedingen, weshalb diese Unterscheidung für den Einzelnen von großer Bedeutung sein kann [2]. 2. In Anbetracht der vorhandenen Daten zur Prävalenz delinquenten Verhaltens im Rahmen von Demenzerkrankungen [4] ist anzunehmen, dass die vermeintliche Seltenheit dieses Phänomens wesentlich dadurch bedingt ist, dass die verfügbaren Zahlen aus dem Bundeszentralregister (BZG) oder der polizeiliche Kriminalstatistik (PKS) lediglich die offiziell bekannt gewordene sog. Hellfeldkriminalität abbilden. Längsschnittlich entwickeln mehr als 10 % der Patienten mit einer Alzheimer-Erkrankung und mehr als 50 % aller Patienten mit frontotemporalen Lobärdegenerationen delinquentes Verhalten [4, 5], sodass eine große Dunkelziffer angenommen werden muss. 3. Obwohl herausforderndes Verhalten und Aggressivität bekannte Symptome fortgeschrittener Demenzerkrankungen sind, ist wenig über die eigentliche Prävalenz, Entstehungsmechanismen und Deliktcharakteristika kriminellen Verhaltens im Rahmen von Demenzerkrankungen bekannt. Die Grenzen zwischen nichtkriminellen und kriminellen Verhalten sind dabei fließend und nicht selten auch dadurch bedingt, dass beim Demenzpatienten nicht jedes aggressive Verhalten zur Anzeige kommt. In der vorhandenen Literatur wird kriminelles Verhalten in diesem Zusammenhang als ein Verhalten definiert, welches gegen geltende Gesetze verstößt. Für den behandelnden Arzt ist die grundlegende Kenntnis dieser Aspekte jedoch auch klinisch relevant.

## Alterskriminalität – allgemeine Aspekte und aktuelle Entwicklungen

**Figure 1:**
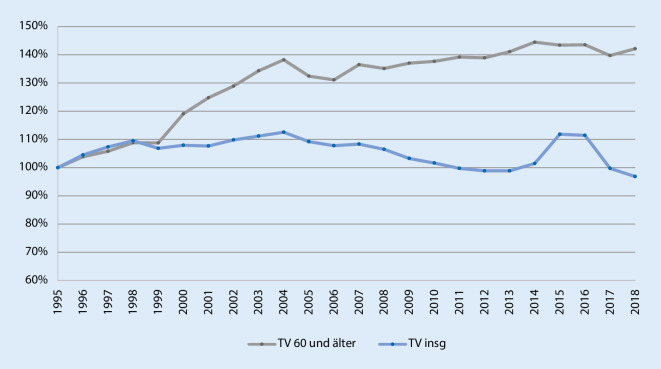

**Figure 2:**
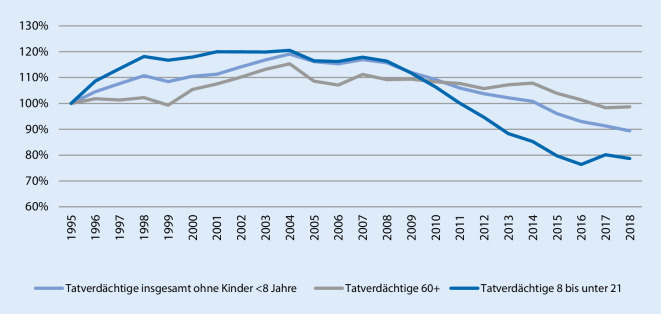

Aus gerontopsychiatrischer Sicht ergibt sich zunächst eigentlich kein Grund, sich an dieser Stelle mit gesunden Älteren zu befassen, hätte nicht der Bundesgerichtshof (BGH) wiederholt zu diesem Thema Stellung bezogen [2]. In mehreren Urteilen rät der BGH mittlerweile grundsätzlich zu Zweifeln an der Schuldfähigkeit von Älteren. Subklinische Veränderungen wie Beeinträchtigungen des Hemmungsvermögens und eingeschränkte Verdrängungsmechanismen werden hier ins Feld geführt [2]. Vor diesem Hintergrund und aufgrund des Kontinuums von physiologischem kognitivem Altern zu krankhaften Abbauprozessen ist es sinnvoll, zunächst die gesamte Seniorenkriminalität zu betrachten. Man bedient sich hierfür der PKS, einer systematischen und kontinuierlichen Bestandsaufnahme von Kriminalität in Deutschland, die systematisch Tatverdächtige erfasst und jährlich vom Bundeskriminalamt veröffentlicht wird. Die wesentliche Limitation der PKS ist, dass die Zahlen in hohem Maße von der polizeilichen Aufklärungsquote abhängen. Die PKS bildet somit ebenfalls lediglich die sog. Hellfeldkriminalität ab. 2018 ermittelte die PKS in Deutschland 2.051.266 Tatverdächtige, davon 155.832 Tatverdächtige ≥ 60 Jahre [6]. Bei der Betrachtung der Entwicklung der Anzahl der Tatverdächtigen entsteht zunächst der Eindruck einer Zunahme der Seniorenkriminalität (Abb. 1). Dazu ist jedoch anzumerken, dass 2018 bereits 28 % der deutschen Bevölkerung > 60 Jahre alt waren, die über 60-Jährigen unter den Tatverdächtigen jedoch mit lediglich 7,6 % repräsentiert waren [6]. Auch die Betrachtung der Tatverdächtigenbelastungszahl (TVBZ: Anzahl Tatverdächtige einer Altersgruppe/100.000 Einwohner dieser Altersgruppe) illustriert, dass Seniorenkriminalität weitgehend dem allgemeinen Trend rückläufiger Kriminalitätszahlen folgt (Abb. 2). Senioren haben insgesamt eine geringe Kriminalitätsbelastung [1]. Als mögliche Ursachen dafür werden die häufig häuslich strukturierten Freizeitaktivitäten, somatische Limitationen durch Multimorbidität, eine erhaltene soziale Kontrolle durch die Familie und ein gewisser Konservatismus mit geringer Motivation zu delinquentem Verhalten angesehen [1].

**Figure 3:**
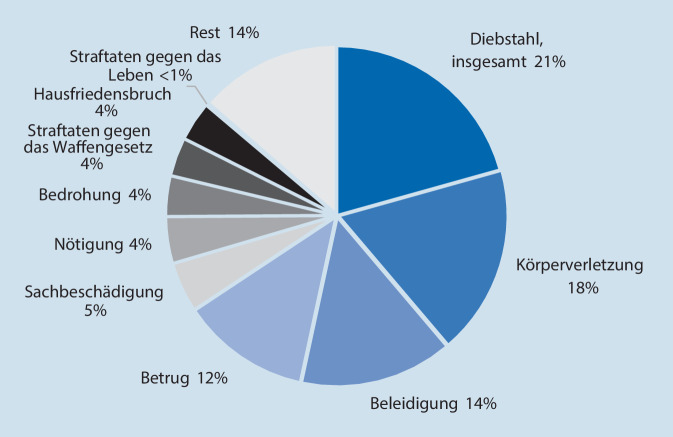

Welche Straftaten 2018 von Senioren verwirklicht wurden ist in Abb. 3 dargestellt [6]. Hierbei zeichnet sich das Bild einer Mischung aus Bagatell- und Überforderungskriminalität. Senioren begehen zumeist leichte Straftaten, was wesentlich auf körperliche Limitationen zurückzuführen sein dürfte. Die häufigsten Bagatelldelikte stellen Diebstahl- und Beleidigungsdelikte dar. Hierbei handelt es sich um zwei Deliktarten, die insbesondere bei Beeinträchtigungen des Hemmungsvermögens wahrscheinlicher werden. Bezüglich der Überforderungskriminalität sind zuvorderst die häufig fahrlässigen Körperverletzungen im Rahmen schuldhaft durch Senioren verursachter Verkehrsunfälle zu nennen [1]. Insgesamt ist Seniorenkriminalität ein männlich dominiertes Phänomen. Etwa 75 % aller Tatverdächtigen ≥ 60 Jahre sind männlich [7]. Ferner hat es die Justiz hier in hohem Maße mit Erstkriminalität zu tun. Von den über 60-Jährigen Ladendieben sind 85–91 % Ersttäter [7].

## Delinquenz im Rahmen von Demenzerkrankungen

Insbesondere hinsichtlich Erstdelinquenz im höheren Lebensalter werden Demenzerkrankungen in der Literatur als bedeutsam eingeschätzt. Doch wie häufig sind Demenzerkrankungen tatsächlich Ursache von Erstkriminalität im höheren Lebensalter? Und welche Erklärungsmodelle zu delinquentem Verhalten im Rahmen von Demenzerkrankungen existieren? Um derartige Fragestellungen zu untersuchen, eignen sich vor allem ältere Ersttäter, zu denen jedoch erstaunlich wenig Literatur existiert. Oft wird in entsprechenden Studien nicht zwischen Erst- und Wiederholungstätern differenziert. Gerade einmal eine Studie aus dem Großraum Tel Aviv untersuchte 28 Ersttäter ≥ 65 Jahre und ermittelte dabei eine Demenzprävalenz von 21 % [8]. Nach aktuellen Erkenntnissen geht man heute davon aus, dass kriminelles Verhalten im Rahmen von Demenzerkrankungen durch früh im Erkrankungsverlauf auftretende Defizite im Bereich der sozialen Kognition, durch Beeinträchtigungen der Fähigkeit zur moralischen Bewertung von Situationen und durch Enthemmungsphänomene begünstigt wird [9]. Diese Teilaspekte werden in der Literatur häufig als „erworbene Soziopathie“ oder „Pseudopsychopathie“ zusammengefasst [9]. Gemäß ICD-10-Klassifikation sind diese Verhaltensänderungen als organische Persönlichkeitsstörung (ICD-10: F07.0) zu kodieren. Als Modellerkrankung gilt hierbei die frontotemporale Demenz, die durch eine frühe Manifestation sozial inadäquaten und häufig moralisch und legal normüberschreitenden Verhaltens gekennzeichnet ist [10]. Darüber hinaus sind die im Erkrankungsverlauf häufiger werdenden Verhaltensstörungen wie Aggressivität und Impulsivität sowie seltene Wahnsyndrome wie das Capgras-Syndrom und der meist hirnorganisch bedingte Eifersuchtswahn (sog. Othello-Syndrom) möglicher psychopathologischer Ursprung von Gewaltdelikten im höheren Lebensalter [2].

## Prävalenz von Demenzerkrankungen in forensischen Kohorten älterer Straftäter

Wie häufig sind Demenzerkrankungen in forensischen Kohorten älterer Straftäter? Hierzu existieren überwiegend retrospektive Querschnittsstudien von psychiatrischen Sachverständigengutachten mit eher deskriptivem Charakter, die jedoch erhebliche methodische Probleme aufweisen. Diese Studien differenzieren in aller Regel nicht nach Erst- und Wiederholungstätern, unterscheiden selten verschiedene Demenzätiologien und untersuchen mitunter verschiedene Altersgruppen, was erhebliche Prävalenzunterschiede bedingt. Darüber hinaus wird ein Gutachtenauftrag lediglich bei begründetem Verdacht auf eine bestehende Psychopathologie erteilt, was eine Positivselektion bedingt. Ferner sind Prävalenzunterschiede auch dadurch bedingt, dass die Schwelle zur forensisch-psychiatrischen Evaluation länderspezifisch erheblich variiert. Nachfolgend werden die Ergebnisse von vier repräsentativen Studien dargestellt, die die Analyse der Prävalenz von Demenzerkrankungen und forensischen Kohorten älterer Straftäter zum Gegenstand hatten und gleichzeitig die damit verbundenen methodischen Schwierigkeiten verdeutlichen.

Lewis und Kollegen untersuchten 99 Straftäter > 60 Jahre, die aufgrund gewaltsamer und nichtgewaltsamer Delikte verhaftet und zur Beurteilung von Schuld- oder Verhandlungsfähigkeit einer forensisch-psychiatrischen Begutachtung zugeführt wurden [11]. Dabei wurde eine überraschend hohe Prävalenz von 44,4 % nicht näher spezifizierter Demenzerkrankungen ermittelt. Es ist anzumerken, dass es sich hierbei um komplex kardiovaskulär erkrankte Probanden mit insgesamt unterdurchschnittlichem Bildungsniveau handelte, die zu 67 % alkoholabhängig waren und somit a priori ein komplexes Demenzrisikoprofil aufwiesen. Die Arbeit schien die häufig postulierte hohe Bedeutung von Demenzerkrankungen im Bereich der Erstdelinquenz zu relativieren, da es sich bei 80,8 % um Wiederholungstäter handelte. Ferner deutete diese Studie auf die besondere forensisch-psychiatrische Bedeutung paranoider Symptome im Rahmen von Demenzerkrankungen hin. 31,3 % der Straftäter wiesen zum Tatzeitpunkt paranoides Erleben auf und davon 22,6 % im Rahmen einer Demenzerkrankung.

Eine schwedische Analyse von 7297 forensischen Sachverständigengutachten beinhaltete 210 Gutachtenprobanden > 60 Jahre und 103 Gutachtenprobanden > 65 Jahre [12]. Dabei wurde eine Gesamtprävalenz von Demenzerkrankungen von 0,3 % ermittelt. Diese vergleichsweise deutlich geringere Prävalenz ist dadurch begründet, dass in Schweden überwiegend Straftäter einer forensisch-psychiatrischen Evaluation zugeführt werden, die schwere Delikte wie Mord oder Vergewaltigung mit meist haftgebundenen Strafmaßen verwirklicht haben. Denn derartige Delikte werden insbesondere von älteren Straftätern seltener begangen. Doch auch bei diesen Probanden ist ein Anstieg der Demenzprävalenz auf 7 % bei > 60-Jährigen und auf 9 % bei > 65-Jährigen zu beobachten.

Eine Arbeit aus China untersuchte eine Kohorte von 4484 Probanden mit insgesamt 47 dementen Straftätern [13]. Somit wurde in dieser Studie eine Demenzprävalenz von 1 % bei recht heterogener Deliktschwere (42,6 % Diebstahldelikte, 12,8 % Mord/fahrlässige Tötung) ermittelt. Erwartungsgemäß wurde den dementen Straftätern in der Mehrzahl (91,5 %) eine relevant verminderte Schuldfähigkeit attestiert (63,8 % verminderte, 27,7 % aufgehobene Schuldfähigkeit). Ferner verdeutlichte die Studie die hohe Verantwortung forensischer Gutachter in diesen Belangen. Wie auch in anderen Studien zu diesem Thema stimmte die richterliche Würdigung der Schuld(un)fähigkeit in 91,5 % der Fälle mit der forensisch-psychiatrischen Einschätzung überein.

Eine andere Analyse ermittelte hingegen eine überraschend geringe Demenzprävalenz von 0,01 % in einer südkoreanischen Kohorte älterer Straftäter, die einer forensisch-psychiatrischen Begutachtung unterzogen worden waren [14]. Dazu ist anzumerken, dass es sich hierbei zum einen um eine verhältnismäßig junge Kohorte handelte und zum anderen, dass das verwendete kognitive Screening, bestehend aus Schädel-MRT, EEG und Mini Mental Status Test (MMST), nicht den heutigen Sensitivitätsansprüchen an die Demenzdiagnostik genügt.

Diese Studien verdeutlichen zusammenfassend die methodischen Schwierigkeiten bei der Schätzung der Demenzprävalenz in forensischen Kohorten älterer Straftäter und die damit verbundenen erheblichen Schwankungen der Prävalenz in derartigen Studienpopulationen. Zusammenfassend ergeben sich die erheblich variierenden Demenzprävalenzen in den verfügbaren Studien durch Altersunterschiede der untersuchten Kohorten, länderspezifisch verschiedene Zugangsschwellen zur forensisch-psychiatrischen Evaluation und nicht zuletzt auch durch verschiedene Sensitivitäten der verwendeten kognitiven Assessments.

## Neurobiologie delinquenten Verhaltens

Im Zeitalter vor struktureller, metabolischer und funktioneller Bildgebung wurden die Zusammenhänge zwischen Hirnstruktur und Hirnfunktion meist anhand von Fällen mit traumatischen Hirnläsionen beschrieben. Hinlänglich bekannt ist die Geschichte von Phineas Gage – einem Gleisarbeiter, der 1848 während Gleisarbeiten eine penetrierende Hirnverletzung im Bereich beider Frontallappen erlitt und nachfolgend eindrückliche Persönlichkeitsveränderungen im Sinne eines infantilen, impulsiven und unzuverlässigen Verhaltens entwickelte [15]. Neurowissenschaftlich war die erlittene Hirnläsion interessant, da sie darauf hindeutete, dass das Frontalhirn für Prozesse wie soziale Kognition und Verhaltenssteuerung zuständig ist. Die erlittene Hirnläsion wurde aus diesem Grund neuroanatomisch genauer kartiert [15]. Demnach erlitt Gage Läsionen im Bereich des linken Orbitofrontalkortex, im Bereich des ventromedialen Präfrontalkortex beidseits und im Bereich des anterioren Gyrus cinguli. Diese Hirnregionen gehören nach heutigem Verständnis zu einem neuronalen Netzwerk, welches beispielsweise bei der Konfrontation mit persönlichen moralischen Dilemmata aktiviert wird, wo der eigene Nutzen gegen den möglichen Schaden anderer abgewogen werden muss [16, 17]. Außerdem vermitteln diese Hirnregionen prosoziale Kompetenzen wie Fairness, Empathie und Prozesse der sozialen Kognition [16, 17].

Nach heutigem Verständnis entsteht delinquentes und unmoralisches Verhalten im Rahmen von Demenzerkrankungen durch die Beeinträchtigung dreier Kognitionsbereiche [16]. Zuvorderst bedeutsam sind Defizite im Bereich der sozialen Kognition, also beispielsweise der Fähigkeit, subtile soziale Signale und Gesten wie Blicke, Mimik und Körperhaltung zu dechiffrieren. Die dafür relevanten Hirnstrukturen sind die Amygdala, der Sulcus temporalis superior posterior und temporoparietale Verbindungen. Darüber hinaus sind Beeinträchtigungen der Verhaltens- bzw. Impulskontrolle bedeutsam, die im Wesentlichen durch Schädigungen im Bereich des Gyrus frontalis inferior und des medialen sowie inferioren Orbitofrontalkortex bedingt sind. Ferner relevant sind Beeinträchtigungen der emotionalen Empathiefähigkeit, die durch Funktionsstörungen im Bereich des anterioren Gyrus cinguli und der vorderen Inselregion vermittelt werden. Daneben ist der ventromediale Präfrontalkortex für die emotionale Empathiefähigkeit wichtig, der aber gleichzeitig auch für Teilaspekte der sozialen Kognition bedeutsam ist [16]. Diese neuroanatomischen Zuordnungen verdeutlichen, weshalb insbesondere neurodegenerative Prozesse im Bereich frontaler, aber auch anteriorer temporaler Hirnareale zu Persönlichkeitsänderungen prädisponieren und delinquentes Verhalten wahrscheinlicher machen.

Eine Arbeit, die die vorangestellten Aspekte verdeutlicht, untersuchte 17 Probanden, bei denen ein eindeutiger Zeitzusammenhang zwischen erlittener Hirnläsion und Erstdelinquenz bestand [17]. Diese Probanden hatten mehrheitlich schwere Gewaltdelikte nach Hirnläsionen im Bereich des orbitalen und ventromedialen Präfrontalkortex sowie in verschiedenen Bereichen des Temporallappens verwirklicht. Zunächst verwunderten hier die insgesamt heterogenen Lokalisationen der verschiedenen Hirnläsionen. Über eine sog. Läsionsnetzwerkkartierung untersuchten die Autoren jedoch, mit welchen Hirnregionen die Läsionen funktionelle Verbindungen aufweisen. Dabei sah man, dass all diese Läsionen funktionell über ein neuronales Netzwerk miteinander verbunden sind. Anhand von fMRT-Datenbanken konnte man nachweisen, dass dieses neuronale Netzwerk große Überschneidungen zu Hirnregionen zeigt, die für Teilkomponenten moralischer Entscheidungsfindung wie Theory-of-mind-Prozesse verantwortlich sind – also der Fähigkeit, den affektiven und kognitiven Status des Gegenübers zu mentalisieren. Aus diesem Grund spricht man von einem neuromoralischen Netzwerk, zu welchem die schon genannten Strukturen Orbitofrontalkortex, ventromedialer Präfrontalkortex und anteriorer Temporallappen gehören. Dies verdeutlicht die hohe Bedeutung frontaler und anteriorer temporaler Hirnstrukturen bei der Entwicklung von Delinquenz im Rahmen traumatischer Hirnläsionen und neurodegenerativer Demenzerkrankungen.

Darüber hinaus bestehen Assoziationen zwischen aggressivem und gewalttätigem Verhalten im Rahmen von Verhaltensstörungen bei Demenzerkrankungen und verschiedenen Manifestationen kriminellen Verhaltens [9]. Zu den Faktoren, die das Risiko für gewalttätiges Verhalten im Rahmen von Demenzerkrankungen erhöhen, zählen insbesondere psychotische und depressive Symptome sowie ein regelmäßiger Alkoholkonsum [9]. Bei der Entwicklung kriminellen Verhaltens im Rahmen der frontotemporalen Demenz sind vordergründig Defizite im Bereich der Empathiefähigkeit („theory of mind“) von Bedeutung [18]. Aus diesem Grund begehen FTD-Patienten häufig Delikte, deren Entstehung im Rahmen antisozialer Persönlichkeitsveränderungen in Kombination mit Enthemmungsphänomenen erklärbar sind [18]. Im Gegensatz dazu sind Delikte im Rahmen einer Demenz vom Alzheimer-Typ seltener gewalttätiger oder aggressiver Natur, sondern eher Folge kognitiver Defizite [18].

## Diagnostische Aspekte

In der Folge soll nun erläutert werden, inwieweit die vorangestellten Aspekte zu Persönlichkeits- und Wesensänderungen im Rahmen von Demenzerkrankungen heute in der Demenzdiagnostik berücksichtigt werden, da dies insbesondere in der forensischen Begutachtungssituation bedeutsam ist. Zunächst verwundert es nicht, dass Defizite der sozialen Kognition bei neurodegenerativen Erkrankungen kognitiv-mnestischen Defiziten um Jahre vorausgehen können, da die soziale Kognition intakte Auffassungs- und Wahrnehmungsleistungen, unbeeinträchtigte Exekutivfunktionen und letztlich auch ein intaktes Gedächtnis voraussetzt und somit einen kognitiven Prozess höherer Ordnung darstellt [19]. In den aktuellen diagnostischen Klassifikationssystemen sind Defizite der sozialen Kognition mittlerweile zentrales diagnostisches Merkmal von Demenzerkrankungen. Aber auch im Bereich der Frühdiagnostik finden Wesens- und Persönlichkeitsänderungen zunehmend Berücksichtigung. Hier vollzieht sich in den letzten Jahren ein gewisser Wandel. Standen insbesondere in der klinischen Alzheimer-Forschung in den letzten Jahrzehnten prodromale und präklinische Symptome wie leichtgradige oder subjektive Neugedächtnisstörungen und deren prädiktiver Wert im Vordergrund, widmet man sich zunehmend Wesensänderungen und deren diagnostischer Bedeutung. Hier wurde mit dem MBI („mild behavioral impairment“) mittlerweile ein diagnostisches Konstrukt geschaffen, welches die Erstmanifestation wesensuntypischer Verhaltens- und Persönlichkeitsänderungen nach dem 50. Lebensjahr beschreibt [20]. Neuropsychiatrische Symptome wie Depressivität, Angst, Euphorie und Irritabilität treten sowohl bei kognitiv gesunden Älteren als auch in Prodromalstadien von Demenzerkrankungen auf [21]. Eine große diagnostische Herausforderung ist jedoch die Differenzierung zwischen prämorbiden psychiatrischen Symptomen und psychopathologischen Auffälligkeiten, die sich erst im höheren Lebensalter manifestieren, was eine relevante Limitation hinsichtlich Spezifität und prädiktivem Wert des MBI darstellt [21]. Insbesondere die affektive und die emotionale Dysregulation sind häufige neuropsychiatrische Symptome im Rahmen präklinischer und prodromaler Demenzsyndrome und sind mit einem progredienten kognitiven Abbau vergesellschaftet [21]. Das MBI gilt heute als Risikofaktor für die Entwicklung einer frontotemporalen Demenz sowie für eine Demenz vom Alzheimer-Typ [20]. Dieses Konstrukt bedarf zwar aktuell einer weiteren prospektiven Validierung, fasst aus forensisch-psychiatrischer Sicht jedoch ein hochrelevantes psychopathologisches Syndrom zusammen, von welchem der psychiatrische Sachverständige Kenntnis haben sollte. Für die forensisch-psychiatrische Praxis bedeutet es, dass bei neu aufgetretenen, wesensuntypischen Persönlichkeitsveränderungen jenseits des 50. Lebensjahres eine neurodegenerative Genese ins Kalkül gezogen werden sollte und dass in derartigen Situationen proaktiv nach solchen, oft subtilen psychopathologischen Veränderungen gefahndet werden muss. Diese sollten ausführlich exploriert und, wenn im forensisch-psychiatrischen Kontext möglich, unter Berücksichtigung des Zeugnisverweigerungsrechts auch fremdanamnestisch erfragt werden.

## Schuldfähigkeitsbegutachtung

**Figure 4:**
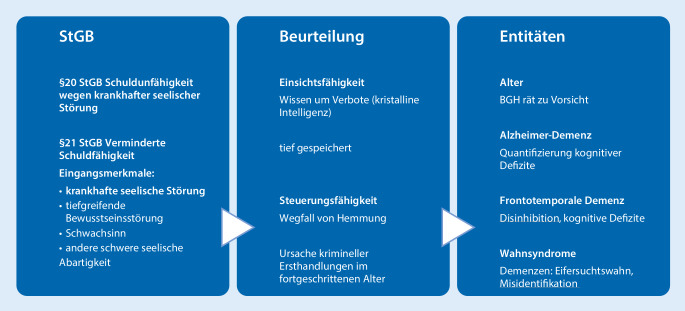

Schuldfähigkeit ist im Strafgesetzbuch (StGB) negativ definiert. Das heißt, es werden Ausnahmen bzw. sog. Eingangsmerkmale aufgeführt, welche Einschränkungen der Schuldfähigkeit bedingen können ([2]; Abb. 4). Das für die Begutachtung von Demenzerkrankungen relevante Eingangsmerkmal des § 20 StGB ist die „krankhafte seelische Störung“. Wenn das Eingangsmerkmal berührt ist, wird in einem zweiten Schritt dann normativ bewertet, inwieweit das diagnostizierte Störungsbild Auswirkungen auf die Einsichts- und Steuerungsfähigkeit begründet [2]. Bei leichtgradigen Demenzerkrankungen ist die Einsichtsfähigkeit häufig unbeeinträchtigt. Einsichtsfähigkeit fasst hier das meist tief gespeicherte Wissen um Verbote zusammen und ist somit den kristallinen Intelligenzfunktionen zuordenbar, welche relativ robust gegenüber kognitivem Altern und auch gegenüber beginnenden krankhaften Abbauprozessen sind [2]. Bei der Begutachtung dementer Straftäter bedeutsamer sind Beeinträchtigungen der Steuerungsfähigkeit, da Einschränkungen des Hemmungsvermögens häufig psychopathologischer Ursprung von Delikten sind. Während der BGH bei Hochbetagen grundsätzlich zu Zweifeln an der unbeeinträchtigten Schuldfähigkeit rät, geht es bei Probanden mit einer Demenz vom Alzheimer-Typ wesentlich um die Quantifizierung des kognitiven Defizitsyndroms und bei Straftätern mit frontotemporaler Demenz um die Feststellung des Ausmaßes der Disinhibition [2]. Zu würdigen sind außerdem hirnorganisch bedingte Wahnsyndrome wie der Eifersuchtswahn oder die seltenen Missidentifikationssyndrome als möglicher psychopathologischer Ursprung von Delikten im höheren Lebensalter, da diese in aller Regel in eine Exkulpierung münden.

## Forensische Besonderheiten verschiedener Demenzätiologien

Unter den neurodegenerativen Demenzerkrankungen prädisponiert insbesondere die frontotemporale Demenz zu moralisch normüberschreitendem und delinquentem Verhalten. 37–57 % aller Patienten mit frontotemporaler Demenz entwickeln im Erkrankungsverlauf kriminelles Verhalten [4], während sie ein verhältnismäßig breites Spektrum an Delikten zeigen. In besonderer Häufigkeit begehen diese Patienten Diebstahl‑, Verkehrs‑, Sexual- und Gewaltdelikte [3]. Doch auch bei der semantischen Demenz, einer diagnostischen Entität der frontotemporalen Lobärdegenerationen (FTLD) (gleichzusetzen mit der semantischen Variante der primär progressiven Aphasie; svPPA), kommt es längsschnittlich wesentlich häufiger zur Delinquenzentwicklung (21–55 %) als bei Patienten mit einer Demenz vom Alzheimer-Typ (5–12 %) [4]. Während Patienten mit semantischer Demenz typischerweise Diebstahl- und Verkehrsdelikte verwirklichen, zeichnen sich Patienten mit Alzheimer-Demenz überwiegend durch leichtgradige Verkehrs- oder Diebstahldelikte im Rahmen einer Überforderungskriminalität bei ausgeprägteren kognitiven Defiziten aus [3]. Klassische Beispiele sind hier das Vergessen, Rechnungen zu bezahlen, oder schuldhaft verursachte Verkehrsunfälle in unübersichtlichen Verkehrssituationen.

Eine retrospektive Kohortenstudie von Liljegren und Kollegen aus dem Jahr 2015 zeigte, dass sowohl Patienten mit frontotemporaler als auch mit semantischer Demenz in signifikant höherem Maße zu delinquentem Verhalten neigen als Patienten mit Alzheimer-Demenz [3]. Diese Studie illustrierte außerdem, dass 14 % aller Patienten mit frontotemporaler Demenz Delinquenz als Erstsymptom aufweisen (frontotemporale Demenz 14 % vs. Alzheimer-Demenz 2 %; *p* < 0,001) und dass diese Patienten im Vergleich zu Patienten mit Alzheimer-Demenz signifikant häufiger zu Gewalttätigkeit neigen (frontotemporale Demenz 6,4 % vs. Alzheimer-Demenz 2 %; *p* = 0,003). Eine weitere retrospektive Kohortenstudie von Liljegren und Kollegen aus dem Jahr 2019 zeigte, dass nicht nur delinquentes, sondern auch sozial inadäquates Verhalten bei Patienten mit frontotemporaler Demenz signifikant häufiger ist als bei Patienten mit Alzheimer-Demenz (frontotemporale Demenz 74,8 % vs. Alzheimer-Demenz 56,4 %; *p* = 0,004; [5]). Darüber hinaus zeigte diese Studie auch, dass über 80 % der Patienten mit frontotemporaler Demenz nach einem Erstdelikt mindestens ein weiteres begehen. Eine Besonderheit dieser Untersuchung war, dass die klinischen Diagnosen der Patienten post mortem neuropathologisch bestätigt wurden. In diesem Rahmen beschrieben die Autoren eine Assoziation zwischen kriminellem Verhalten bei Patienten mit frontotemporaler Demenz mit Non-Tau-Pathologie. Diesbezüglich ist jedoch anzumerken, dass in der frontotemporalen Demenzgruppe mit Tauopathie auch andere Tauopathien wie kortikobasale Degenerationen (CBD) und Fälle mit progressiven supranukleären Blickparesen (PSP) vertreten waren, die primär durch eine mittelliniennahe Neurodegenration und weniger durch eine präfrontale Beteiligung charakterisiert sind. Die Autoren schlussfolgerten, dass Delinquenz wahrscheinlich weniger eine Funktion der Art, sondern vielmehr der Lokalisation des abgelagerten Proteins ist. Diehl-Schmidt und Kollegen zeigten anhand einer Angehörigenbefragungsstudie ebenfalls, dass sämtliche Deliktarten bei Patienten mit frontotemporalen Lobärdegenerationen häufiger vorkommen als bei Patienten mit Alzheimer-Demenz [22]. Interessanterweise fanden die Autoren keinen Häufigkeitsunterschied hinsichtlich delinquenten Verhaltens zwischen Patienten mit frontotemporaler und semantischer Demenz. Dies ist am ehesten dadurch erklärbar, dass sich die semantische Demenz, die klinisch primär durch Benennstörungen und den progredienten Verlust von Objektwissen charakterisiert ist, histopathologisch primär durch eine Neurodegeneration des anterioren Temporallappens und damit auch der Amygdala, einem Zentrum für soziale Kognition, auszeichnet [16]. Dies lässt annehmen, dass delinquentes Verhalten bei Patienten mit semantischer Demenz am ehesten als Folge sozialkognitiver Defizite zu werten ist. Der einzige soziodemografische Unterschied zwischen FTLD-Patienten mit und ohne kriminellem Verhalten bestand in der untersuchten Kohorte in der Erkrankungsdauer, was als weiterer Hinweise auf eine hohe längsschnittliche Prävalenz delinquenten Verhaltens im Rahmen frontotemporaler Demenzerkrankungen gedeutet werden kann. Mendez und Kollegen befragten Patienten mit frontotemporaler Demenz zu verwirklichten Delikten [10] und ermittelten, dass die Patienten mit frontotemporaler Demenz in aller Regel um ihr Vergehen wissen und auch angeben, es zu bereuen, jedoch ohne dass adäquate Emotionen wie Scham oder Reue greifbar werden. Die Autoren schlussfolgerten, dass delinquente Patienten mit frontotemporaler Demenz zwar über ein erhaltenes faktisches Schuldbewusstsein verfügen, jedoch gravierende Beeinträchtigungen des emotionalen Schuldbewusstseins zeigen. Dieses Phänomen verdeutlicht die Herausforderung für forensisch-psychiatrische Sachverständige bei der Konfrontation mit Gutachtenprobanden, die formal uneingeschränkt einsichtsfähig sind, Delikte jedoch im Rahmen von Wesensänderungen als Krankheitssymptom begangen haben und keine adäquate Emotion wie Reue oder Scham zeigen. Die längsschnittliche Rekonstruktion von – verglichen mit dem prämorbiden Ausgangsniveau verschiedenen – delinquenten Verhaltensbereitschaften und die Diskussion daraus resultierender Fähigkeitsbeeinträchtigungen mit Tatbezug entsprechend §§ 20, 21 StGB, v. a. der tatbezogenen exekutiven Steuerungsfähigkeit, sind demnach wesentliche Aufgaben des forensisch-psychiatrischen Sachverständigen, wozu das Wissen um aktuelle Konzepte und Kriterien von Demenzerkrankungen unverzichtbar ist.

## Zusammenfassung und Ausblick

Alterskriminalität gilt als seltenes Phänomen, wobei insgesamt von einer großen Dunkelziffer ausgegangen werden muss, da ältere Straftäter weniger wahrscheinlich angezeigt und strafrechtlich verfolgt werden. Die Demenzprävalenz in forensischen Kohorten älterer Straftäter ist am ehesten aus studienmethodischen Gründen extrem heterogen und schwankt zwischen 0,01 und 44,4 %. Insbesondere neurodegenerative Prozesse in frontalen und anterioren temporalen Hirnteilen prädisponieren zu delinquentem Verhalten. Zur Erklärung wird das Modell der erworbenen Soziopathie bemüht, welches Defizite im Bereich der sozialen Kognition, spezifische Defizite der Fähigkeit, Situationen moralisch zu bewerten, und eine allgemein verminderte Verhaltenskontrolle syndromal zusammenfasst. Von hoher forensisch-psychiatrischer Relevanz sind frontotemporale Lobärdegenerationen wie die frontotemporale Demenz und die semantische Demenz, da sich delinquentes Verhalten hier früh im Verlauf manifestiert, insgesamt häufig ist, häufiger Gewaltdelikte begangen werden und höhere Rückfallraten bestehen. Erschwerend kommt hinzu, dass die Delikte im Rahmen derartiger Erkrankungen häufig zu einem Zeitpunkt begangen werden, wo noch keine greifbaren kognitiv-mnestischen Defizite bestehen, die Patienten noch um die negativen Konsequenzen ihres Verhaltens wissen, aber bereits Beeinträchtigungen des emotionalen Schuldbewusstseins aufweisen. Insbesondere Delikte im Rahmen wesensuntypischer Persönlichkeitsveränderungen jenseits des 50. Lebensjahres sollten an eine neurodegenerative Ätiologie denken lassen. Aktuelle Daten zeigen, dass insbesondere die Degeneration neuronaler Netzwerke Beeinträchtigungen im Bereich der sozialen Kognition bedingt und wie weitreichend die Folgen der daraus resultierenden Verhaltensänderungen für Betroffene und Angehörige sind. Ein besseres Verständnis dieser Prozesse kann dazu beitragen, Angehörige, Ärzte und auch die Justiz im Umgang mit diesen Verhaltensänderungen und -konsequenzen zu unterstützen. Forschungsbemühungen auf diesem Gebiet können ferner einen wichtigen Beitrag zu einer adäquaten Versorgung der Betroffenen leisten, die in hohem Maße den medizinethischen Grundsätzen entspricht [18]. Insbesondere die frontotemporale Demenz eignet sich aufgrund der prominenten und früh im Erkrankungsverlauf auftretenden Verhaltensänderungen diesbezüglich als Modellerkrankung. Längsschnittliche Beobachtungstudien biologisch und klinisch gut charakterisierter Patienten mit frontotemporaler Demenz können beispielsweise helfen, Assoziationen zwischen spezifischen neuropathologischen Prozessen bzw. Erkrankungsstadien und der Entwicklung kriminellen Verhaltens zu detektieren und die zugrunde liegenden Pathomechanismen besser zu verstehen. Dies könnte auch zur besseren Identifikation von Patienten mit einem hohen Delinquenzrisiko beitragen und würde dadurch einen selektiven Einsatz von Präventionsstrategien ermöglichen.

Ärzte, die mit der Behandlung von Demenzerkrankungen betraut sind, sollten in Anbetracht der längsschnittlich hohen Prävalenz von Demenzerkrankungen über mögliche Verhaltensstörungen mit deliktischer Relevanz aufklären und auch die Entwicklung von Präventionsstrategien als therapeutischen Auftrag annehmen. Entgegen der bisherigen Praxis sollte die biomarkerbasierte Diagnostik insbesondere bei Erstdelinquenz im höheren Lebensalter Einzug in die Diagnostik im Rahmen forensisch-psychiatrischer Begutachtungen halten oder zumindest niedrigschwellig erwogen werden.
